# Multilayer W-doped vanadium dioxide thermal sensors with extended operation region

**DOI:** 10.1016/j.isci.2025.112528

**Published:** 2025-04-24

**Authors:** Callum Wheeler, Yuxiao Zhu, Kai Sun, Bohao Ding, Ruomeng Huang, Otto L. Muskens, C.H. (Kees) de Groot

**Affiliations:** 1 Electronics and Computer Science, Faculty of Engineering and Physical Sciences, University of Southampton, SO17 1BJ Southampton, UK; 2 Physics and Astronomy, Faculty of Engineering and Physical Sciences, University of Southampton, SO17 1BJ Southampton, UK

**Keywords:** Physics, Optics, Materials science

## Abstract

Bolometers rely upon the temperature coefficient of resistance (TCR) of their underpinning sensing layer to detect infrared radiation. Vanadium dioxide (VO_2_) exhibits a very large, but abrupt TCR associated with its monoclinic to rutile phase transition. W-doping of VO_2_ lowers and broadens its transition temperature, and by combining multiple discrete W:VO_2_ layers, a sensing layer can be created with an extended temperature operation region. Herein, we report such a multilayer W:VO_2_ thin film by atomic layer deposition (ALD). The film displays an average TCR of −9.5 (±3.5) %K^−1^ from 30°C to 60°C. A sensing layer consisting of 10 individual W:VO_2_ layers is simulated with a multi-objective genetic algorithm (MOGA) for maximum average TCR (μ) and minimum variation (σ) across an extended target temperature range producing an optimized layer structure at max (|μ|−σ) with average TCR response of −6.7 (±0.9) %K^−1^ from 20°C to 70°C. This work highlights the potential for the broader application of uncooled bolometers.

## Highlights

• W-doped VO_2_ resistivity response model based on experimental ALD film data’
• Experimental thermal sensing performance of multi-layer W:VO_2_ with high TCR
• Multi-objective genetic algorithm optimization of multi-layer W:VO_2_ between 20°C and 70°C
• Performance optimum found for multilayer with flat TCR over the wide temperature range

## Graphical abstract

**Figure:**
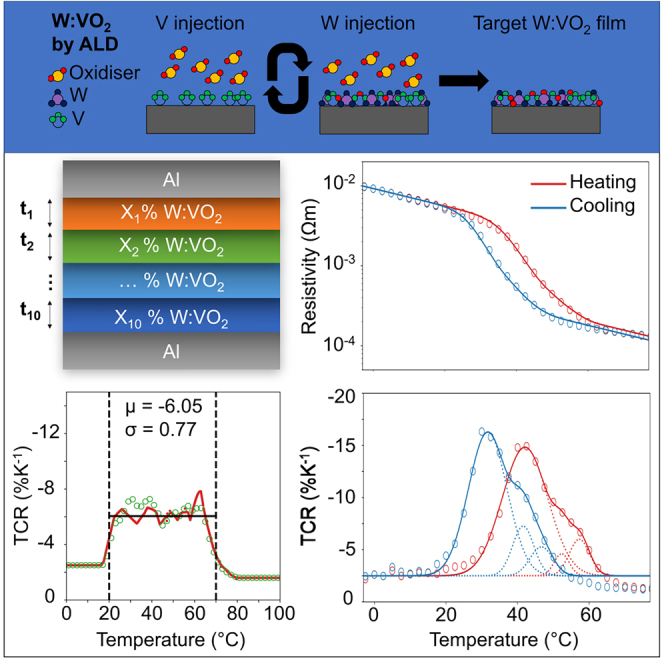

## Introduction

Thermochromic materials have recently seen a spike in interest from the scientific community due to their unique ability to undergo large temperature driven changes in their optical and electrical properties. One such material is vanadium dioxide, which switches reversibly from the insulating monoclinic (VO_2_ (M)) to the metallic rutile phase (VO_2_ (R)) with a characteristic hysteresis around a transition temperature of 68°C. The transition is accompanied by a similar change in optical response between dielectric and metallic, which has application in smart windows,^1^^,^^2^^,^^3^^,^^4^^,^^5^^,^^6^^,^^7^^,^^8^^,^^9^ passive radiative cooling applications,^10^^,^^11^^,^^12^^,^^13^ and infrared identity management.^14^^,^^15^^,^^16^

We will refer in this article, as per convention, to the insulating monoclinic and metallic rutile state of VO_2_, respectively, even though both phases are better described as semiconducting with different free carrier concentrations. The large increase in free charge carriers at the phase transition and the associated large negative temperature coefficient of resistance (TCR) make VO_2_ extremely promising as the active layer in uncooled microbolometers – thermal sensors that measure sample temperature through changes in resistance of the active layer.^17^^,^^18^^,^^19^ These devices are favored compared to their cryogenically cooled counterparts due to their low cost, low power usage, and portability. A wide range of materials have been used to make such devices, either metals (Ti, Pb, Au, and so forth)^20^^,^^21^^,^^22^^,^^23^^,^^24^ or semiconductors (a-Si, Si_x_Ge_y_O_1-x-y_, and so forth).^25^^,^^26^ In general, semiconductors are found as the more promising material due to their higher TCR. A microbolometer figure of merit includes responsivity, noise equivalent power (NEP), and the noise equivalent temperature difference (NEPT). However, for new material development, TCR is widely used to quantify the sensitivity of uncooled microbolometer materials in their early stages and show their viability for commercial applications; thus shall be the focus of this work.

The main disadvantages of VO_2_ are that the phase transition occurs well above room temperature and over a very short temperature range (≈5°C), thus limiting the thermal operating region. To give a greater breadth of applications, precise control of the transition temperature, transition width, and TCR response is imperative. In particular, the reduction of the transition temperature to around room temperature is essential.^27^ This can be achieved by introducing dopant ions, such as W^6+^ and Mo^6+^, reducing the transition temperature.^28^ By doping with a relatively small amount of these ions, a large and predictable decrease in the transition temperature can be achieved. Alternatively, dopants with valences lower than Vanadium can increase the transition temperature.^29^

Previous studies using VO_x_-based bolometer designs have reported very high TCR values over short ranges for planar device architectures.^30^ Materials whose transition occurs over a wider temperature range display lower absolute TCR values.^31^^,^^32^ Device structures, such as V_2_O_5_ nanofibers,^33^ also show enhanced performance compared to typical carbon nanofiber sensors. Recently, a new microbolometer design has been proposed by Guo et al.^34^ in which they utilize the large change of resistance seen in VO_2_ to produce a frequency modulation (FM) based detection technique, differing from the currently available amplitude modulation (AM) based designs. Such a design would benefit greatly from the lower transition temperature of W:VO_2_, leading to a smaller drive current needed for the device’s operation.

Designs utilizing the high TCR phase transition of VO_2_ have shown much greater TCR values compared to their VO_x_ counterparts. By doping the material with a donor metal, the transition temperature and peak absolute TCR value are reduced.^35^^,^^36^ Deposition methods include pulsed laser deposition.^37^^,^^38^^,^^39^ magnetron sputtering^40^^,^^41^^,^^42^; the sol-gel method,^43^^,^^44^^,^^45^ plasma enhanced CVD,^46^ and ALD.^47^ By combining the individual layer responses of undoped VO_2_ and W-doped VO_2_ with various atomic % W, a multilayer design exhibits a high TCR response across a wider temperature range as shown by pulsed laser deposition on crystalline oxides.^37^ ALD provides the best atomic layer thickness control due to its self-limiting nature. Together with its standardized industrial use, this makes it the most suitable deposition technique to design and fabricate such multilayer devices. In this article, we demonstrate the ability of ALD to create highly sensitive VO_2_ thermal sensors with an extended operation temperature region.

We conceptualize and experimentally demonstrate the potential of a multilayer W:VO_2_ thin-film as a high sensitivity thermal detection medium, utilizing the individual thermochromic characteristics of each layer to produce an extended operation region. Five uniformly doped W:VO_2_ thin film samples were fabricated in-house via ALD, giving an experimental group of temperature dependent resistivity responses. A set of 13 parameters was found to accurately predict the resistivity response of these as a function of their at. % W, allowing for the electrical response of W:VO_2_ samples to be predicted up to W = 2.0 at %. As a proof of concept, a multilayer W:VO_2_ design was fabricated via ALD, offering nanometer thickness and high precision W doping control simultaneously. W doping variation through the thin-film was characterized by depth resolved X-ray photoelectron spectroscopy (XPS), with the films showing a clear W distribution and improved electrical performance indicative of multiple W:VO_2_ layers transitioning individually.

To push the limits of this technology, a multi-objective genetic algorithm (MOGA) with Non-dominated Sorting Genetic Algorithms (NSGA-II) technique was used in conjunction with newly modeled W:VO_2_ resistive responses to simulate optimized multilayer W:VO_2_ stacks, generated based upon their mean TCR (*μ*) and the corresponding standard deviation (*σ*) across three temperature ranges. The optimized designs show high TCR values with very low variation across all target temperature windows, providing a method for fast, low-cost material design. Through the excellent control of ALD both in terms of layer thickness and W dopant introduction, the simulated multilayer W:VO_2_ stacks could be manufactured in a commercial environment to the designed specification.

## Results and discussion

### Experimental characterization of W:VO_2_ resistivity response

**Figure 1:**
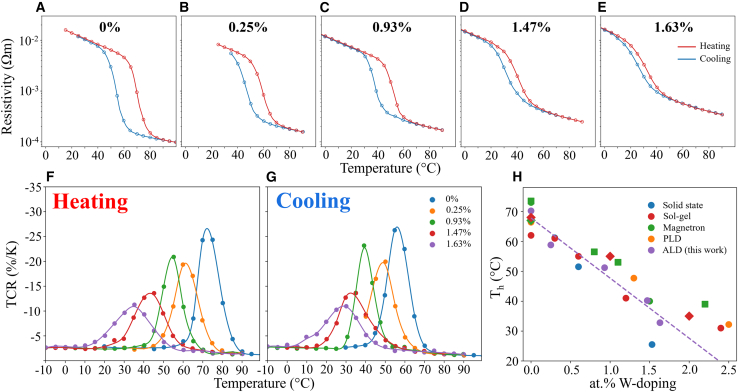
Temperature dependent resistivity of individual W:VO_2_ films Experimental characterization of the electrical response for W:VO_2_ thin films with varied W at.%. (A) 0%, (B) 0.25%, (C) 0.93%, (D) 1.47%, and (E) 1.63%. Hysteresis data are fitted with a second order spline. Experimental TCR response for (F) heating and (G) cooling of all fabricated samples fitted with a second order spline. (H) Extracted T_h_ of varied at.% W:VO_2_ samples prepared through different techniques.^37^^,^^42^^,^^49^^,^^50^^,^^51^^,^^52^^,^^53^ Fit line of our samples presented as the purple dashed line.

Individual W:VO_2_ thin films were grown via ALD with varying concentrations of W. Changing the W/V precursor cycle ratio during deposition results in different doping concentrations, quantified via XPS fitting (ALD growth conditions, ellipsometry, and XPS fitting are available in our previous works).^48^ The W:VO_2_ films were found to have doping levels of 0, 0.25, 0.93, 1.47, and 1.63 at%. Temperature dependent Van der Pauw measurements were taken for all W:VO_2_ samples after annealing, with ellipsometry mapping used to extract the film thicknesses ranging from 30 to 46 nm. Resistivity values were subsequently calculated. Resistivity data for both heating and cooling hysteresis of the varied W at.% samples are plotted in Figures 1A–1E. A change of resistivity of around two orders of magnitude is seen in all samples, with the largest change seen in the undoped VO_2_ thin film. Figures 1F and 1G show the heating and cooling TCR response of the fabricated samples. The extracted transition temperatures for heating runs (*T*_h_) are plotted as a function of W atomic doping percentage in Figure 1H and compared against those reported in the literature. The samples presented here display a linear reduction in *T*_h_ as W dopant levels increase, following the same trend as those manufactured using other deposition techniques in the literature.^14^^,^^49^

It should be noted that there is some variation in the literature around the calculation of TCR, with many articles reporting TCR as α=ΔR/(ΔT×R); the relative change of resistance with respect to temperature per degree. While this is suitable for sets of data with measurements taken in very small temperature steps, for discrete datasets, the equation breaks down due to the logarithmic relation between resistivity and temperature, and so can produce values of α that are lower than the true value. To assess the effective change of resistivity, the TCR was calculated for all data using Equation 1, where ρ and *T* are resistivity and temperature respectively.

(Equation 1) $$\alpha =\frac{dln\left(\rho \right)}{dT}$$

### Multilayer W:VO_2_ for an extended phase transition response

**Figure 2:**
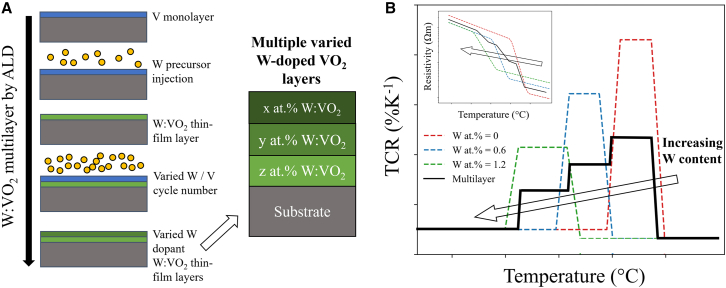
Concept of multilayer W-doped VO_2_ design (A) An example schematic of the ALD fabrication process to produce a multilayer W:VO_2_ stack. By varying the W/V cycle ratio and total cycle number, differing W at.% doping concentrations and layer thickness can be deposited. (B) The benefits of a multilayer design over a single W:VO_2_ layer electrical response with varying W at.%, demonstrating the desired extended phase transition response. The corresponding resistivity responses are also presented as an inset on the figure.

For high accuracy infrared sensing, a large TCR response across the target operating temperature range is required. This operating temperature can be tailored by doping VO_2_ with a high valence donor metal, contributing additional electrons to the material in addition to strain into the lattice.^51^^,^^54^^,^^55^ For a single layer W:VO_2_ stack, the electrical thermochromic response has limited flexibility, offering only two variables for the stack design – layer thickness and doping at.%, with doping defining the operating temperature of the device. To increase the versatility and potential applications of the material, more complex designs with additional parameters need to be produced, offering a tailored thermochromic response. One such design would be a multilayer W-doped VO_2_ stack consisting of multiple discreet W:VO_2_ layers as depicted in Figure 2A, each with a different dopant at.% and thickness, with such control possible via ALD. This design would produce a broadened transition due to the combined thermochromic response of each individual layer.

An example of how such a design could be beneficial for electrical sensing and show improved performance compared to a single W:VO_2_ layer is presented in Figure 2B. The presented multilayer design consists of three individual layers with different transition temperatures defined by their W doping at.%. A large decrease in the stack’s resistivity and associated high TCR response would occur as each layer transitions from its respective monoclinic to rutile phases. By varying the thickness of each individual W:VO_2_ layer, the magnitude of this resistivity change can be controlled.

### Material characterization of multilayer W:VO_2_ devices

To demonstrate the concepts, two multilayer W:VO_2_ devices were fabricated, each targeting differing operating temperature ranges dictated by their dopant percentages and layer thicknesses – one a wide temperature range, high TCR design, and the other a narrow temperature range, very high TCR design. The multilayer was achieved by varying the W and V precursor cycle ratio during the deposition of the stack. A total thickness of around 65 nm was extracted by variable angle spectroscopic ellipsometry for both stacks. Annealing time and oxygen pressure were varied to create optimised structures. X-ray diffraction on the annealed films (see Figure S1) shows the composition of the film to be predominantly VO_2_(M1). The optimized nature of the films is borne out by the electrical measurements in the next section.

**Figure 3:**
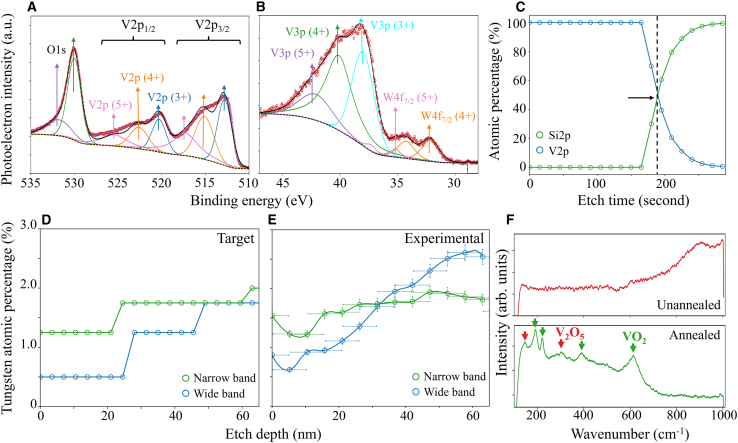
Material characterization of multilayer W:VO_2_ devices XPS spectra of unannealed samples after Ar^+^ ion bombardment for binding energies covering the (A) V2p and (B) V3p binding energy ranges. (C) Relative composition of Si/V as a function of etch time, giving an etch rate of 0.35 nms^−1^ through the unannealed films. The at % W as a function of depth of the wide and narrow temperature range stacks with (D) nominal target as implemented in ALD precursor ratio and (E) experimental W doping depth profile as extracted from XPS W/V ratio. (F) Raman spectra from the pre- and post-annealed stacks, with the main VO_2_ and V_2_O_5_ peaks identified.

Depth resolved XPS measurements were carried out to characterize the W/V compositional variation as a function of depth of the as-deposited multilayer W:VO_2_ stacks. Figures 3A and 3B display an example binding energy spectra covering both the V2p/O1s and V3p/W4f energies, respectively, with each spectrum devolved into their respective electron orbital peaks. The V2p binding energy peaks are conveniently near the O1s peaks within the range 510–535 eV, and so the large O1s peak at 530.0 eV was used to charge correct this spectrum.^45^ There is a shoulder within the O1s peak centered around 531.30 eV corresponding to C=O bonds, indicating that there is some surface contamination. Three doublet V2p peaks with orbital angular momentum of 3/2 and 1/2 corresponding to the V^3+^, V^4+^, and V^5+^ oxidation states were fitted.

To convert the etch time into etch depth, a calibration was performed for each sample by comparing the relative composition of V to Si as a function of etch time, as depicted in Figure 3C. The time at which the ion beam is assumed to have completely etched through the W:VO_2_ thin film is when the relative composition of V and Si at. % are equal. Typically seen within W:VO_2_, the W4f doublet represents most strongly the W^6+^ ionisation.^43^^,^^56^ Under the influence of Ar^+^ ion bombardment, however, to the W^4+^ ionization state becomes the dominant W4f peak shifting due to the preferential sputtering of oxygen.^57^ To determine the W concentration accurately, both the W^6+^ and W^4+^ doublet at. % were used. The target and experimentally extracted at. % W as a function of etch depth for both stacks is presented in Figures 3D and 3E, respectively. Both materials show an increasing at.% W dopant as a function of depth, with the larger ΔW at. % seen in the wide temperature range design as desired. Discreet “steps” in the W concentration are not observed, however, primarily due to limited XPS depth resolution. Nevertheless, these results show that by varying the W/V cycle ratio during deposition, it is possible to achieve tailored W doping through VO_2_ thin films.

Figure 3F shows the Raman spectroscopy spectrum for the as-deposited and optimized annealed sample. The unannealed sample shows no peaks, typical of amorphous VO_2_.^58^ The optimized annealed sample shows strong peaks associated with VO_2_ (M1). The two low energy Raman emission peaks occurring at 192 and 224 cm^−1^ are commonly attributed to A_g_ peaks from V-V bond vibrations in VO_2_ (M1), along with a peak around 615cm^−1^ associated with the V-O A_g_ vibration.^58^^,^^59^ Minor Raman scattering from α-V_2_O_5_ occurs at wavenumbers 145 cm^−1^ and 288 cm^−1^, associated with the V-O B1_g_ vibrational peak.^60^^,^^61^

### Electrical characterization of multilayer W:VO_2_ devices

**Figure 4:**
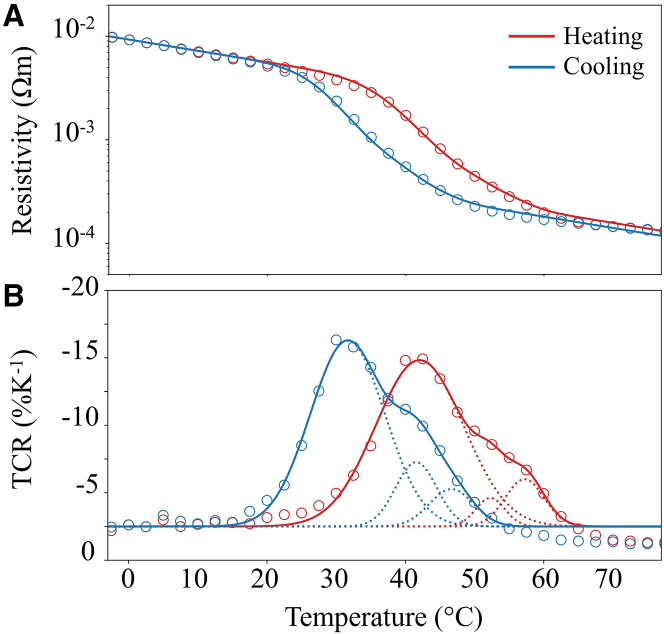
Electrical response of W:VO2 multilayer (A) Experimental resistivity at distinct temperatures (circles), the wide temperature range sample for heating and cooling runs. (B) Associated TCR devolved using three individual Gaussian peaks, taking a uniform baseline of −2.5 %K^−1^. The resistivity and TCR responses predicted from the summed Gaussian peak fits are also shown (solid lines).

Resistivity is a prominent indicator of the material's performance, particularly for sensing materials. The thermochromic electrical performance of the annealed multilayer stacks is presented in Figure 4, along with the associated TCR plot. The annealed samples show a Δρ of around two orders of magnitude, matching well with the previous report of VO_2_ (M1) films deposited upon SiO_2_,^62^ along with a thermal hysteresis characteristic of the material.

Clear characteristics of the multilayer nature of the material can be extracted from the TCR response plotted in Figure 4B. The device shows a broadened electrical phase transition response, quantified by using three Gaussian peaks (dotted lines) and their envelope. As individual layers transition from their insulating to metallic phase, increasing the number of free charge carriers in the material, there is a large decrease in the sample’s overall resistivity, with the magnitude of said TCR response corresponding to the thickness of the transitioning layer. Both heating and cooling runs display a large low temperature TCR peak about 39°C and 28°C, respectively, indicating a W doping of ∼1.5 at% dominating the electrical response. The two smaller TCR peaks are attributed to thinner W:VO_2_ layers with W doping of 0.9 at% and 0.7 at%. The predicted W doping is calculated using Equations 2 and 3 for heating and cooling responses, respectively. The predicted temperature dependent resistivity was calculated and presented in Figure 4A, showing good agreement between experimental and predicted responses verifying the multilayer nature of the sample. This multilayer transition is also verified by optimised anneal studies (Figure S2) and temperature dependent FTIR measurements (Figure S3), further demonstrating the capability of such technology for tailored thermochromic emissivity control.

### Extraction of parameters and modeling resistivity response of W:VO_2_

Due to the high costs of VO_2_ thin-film fabrication via ALD, simulating the electrical response of individual W:VO_2_ as a function of their doping concentration is imperative to produce optimized designs quickly and cheaply. As such, using our uniformly doped single layer experimental data, we create a model to parameterize the resistivity response of W:VO_2_ thin films as a function of their W at.% using a simple three log straight line fit described by TCR_low T_, TCR_max,_ and TCR_high T_. Figure S4 shows an example of the predicted shape for (a) resistivity and (b) TCR values. W:VO_2_ displays a consistent negative (semiconducting) TCR response in both its monoclinic and rutile phases, given by TCR_low T_ and TCR_high T_, respectively. As these responses occur outside the transition region, they are identical irrespective of heating or cooling runs. The resistivity values at 0°C and 100°C ($\rho_{0}$ and $\rho_{100}$) are used as the start and endpoints from which the samples display their monoclinic or rutile TCR responses. The transition temperatures (*T*_c_ and *T*_h_) are defined as the inflection points. A single constant TCR_max_ value now uniquely defines the fit with the transition width defined by the crossing of the TCR_max_ line with the respective TCR_low T_ and TCR_high T_ line, as shown in Figure S4. To represent real resistivity curves more accurately, the TCR value was linearly increased or decreased from ±2.5°C about the node points (positions where the TCR value changes), smoothing the resistivity graphs without adding additional fit parameters.

Our experimentally single layer resistivity data were fitted using this newly defined function as depicted in Figures S5A–S5E for each sample, with the corresponding TCR values plotted beneath (Figures S5F–S5J). This model shows an excellent agreement in both resistivity fitting and subsequent TCR result, with the total integrated area underneath the TCR curves matching between fitted and experimental data.

**Figure 5:**
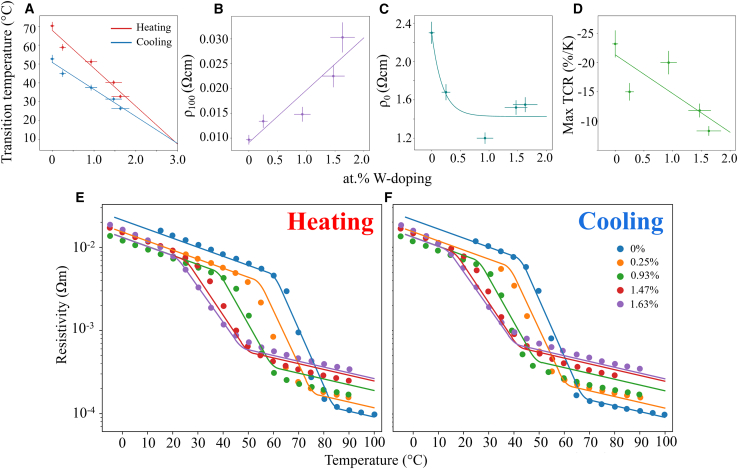
Parameterization of the resistivity response of W:VO2 films Extracted experimental resistivity parameters from the single layer W:VO_2_ films using the model described in this section. Trend plots for (A) the heating ($T_{h}$) and cooling ($T_{c}$) transition temperatures, (B) ρ_0,_ (C) ρ_100_, and (D) the maximum TCR response. Lines are fit to the extracted data with corresponding equations displayed in (Equation 2), (Equation 3), (Equation 4), (Equation 5), (Equation 6), (Equation 7), (Equation 8). Resistivity as a function of W at.% in W:VO_2_ samples using the parametrized model fit (instead of the individual fits) for both (E) heating and (F) cooling runs. Experimental (dots) vs. the model (lines).

The extracted parameters from the deposited W:VO_2_ thin films are presented in Figure 5. Linear trends can be seen in Figure 5A in the heating and cooling transition temperatures and tungsten dopant percentage. As previously discussed, this is a result of the increased lattice strain introduced by the larger W atoms' ionic radii in the crystal lattice among other factors. Our reported decrease in *T*_h_ and *T*_c_ of −20.1 and −14.3 K/at% respectively are consistent with those previously characterized, with *T*_h_ typically −20 K/at%^63^ and *T*_c_ around −14 K/at%.^37^^,^^51^ By extrapolating the fits for both, a dopant amount can be found where both the heating and cooling transition temperature are equal, effectively eliminating the hysteresis commonly found in W:VO_2_. This occurs at ≈2.9 at% W dopant, however, making a material with such a high dopant composition could cause the material to remain in the VO_2_(R) phase across all temperatures due to the increased strain on the crystal lattice^64^ and enhanced charge carrier concentration.^54^

The initial resistivity values at 0°C ($\rho_{0}$) in Figure 5C displays an exponential decay relationship with increased dopant atomic percentages before reaching a saturation value at around 1 at%, after which the material sees no change in this value. With the introduction of W atoms into the VO_2_ crystal lattice, an increase in the density of grain boundaries is commonly reported through AFM measurements,^65^ resulting in higher defect levels in the band gap and increased electron scattering at the grain boundaries.^37^

Shown in Figure 5D, the TCR_max_ undergoes a reduction of 14.9 %K^−1^ between undoped VO_2_ to W = 1.63 at%. The temperature range over which the transition occurs also increases with dopant levels, as seen in the broader experimental TCR peaks. This can be attributed to a decrease in the energy width of the W:VO_2_ transition as predicted using the Looyenga model.^66^^,^^67^ The final 13 parameter fit equations are given later in discussion, where x is the atomic percentage W doping:

(Equation 2) $$T_{h}\;\left({}^{\circ}C\right)=67.8-20.1x$$

(Equation 3) $$T_{c}\;\left({}^{\circ}C\right)=50.8-14.3x$$

(Equation 4) $$\rho_{0}\;\left(\Omega m\right)=1.42\times 10^{-2}+8.77\times 10^{-3}\times e^{-\frac{x}{0.188}}$$

(Equation 5) $$\rho_{100}\;\left(\Omega m\right)=9.10\times 10^{-5}+1.06\times 10^{-4}x$$

(Equation 6) $${TCR}_{\max}\;\left(\%K^{-1}\right)=-21.3+6.63x$$

(Equation 7) $${TCR}_{low\;T}\;\left(\%K^{-1}\right)=-2.5$$

(Equation 8) $${TCR}_{high\;T}\;\left(\%K^{-1}\right)=-1.6$$

With all parameters characterized, the resistivity response of any at.% W doped sample is possible, with the predicted response from this model against the experimental results plotted in Figures 5E and 5F for both heating and cooling runs. The excellent agreement between the experimentally data and the parametrized model means that interpolation and limited extrapolation to x = 2.0 is allowed, providing a modelized W-doped VO_2_ model which can be used as input for simulations.

### Optimized simulated response of multilayer W:VO_2_ bolometer stacks

Using (Equation 2), (Equation 3), (Equation 4), (Equation 5), (Equation 6), (Equation 7), (Equation 8), the resistivity response of any at.% W-doped W:VO_2_ thin film up to approximately 2.0 at.% can be predicted. A numerical simulation was created using Spyder (Python 3.9) in which a 10 layer W:VO_2_ stack design electrical response can be estimated by assuming each layer acts as a resistor in parallel, with the thickness and W doping at. % of each layer as variable parameters. Such a design could be fabricated with atomic layer precision using ALD, and subsequently depositing metallic contacts on the sides of the stack. Alternative design choices were also simulated for resistors in series, predicting the response of alternative geometries such as those seen within graded W:VO_2_ nanotubes. Considering the practical limitations of ALD doping control, the W:VO_2_ materials were limited to W doping values from 0 to 2.0 at. %. in 0.05 at. % steps, with the thickness of each resistor limited from 3 to 100 nm and total thickness of 500nm. This design limitation can be realized by ALD in a commercial setting with neither time nor costs being unreasonable for an uncooled microbolometer by using optimized large area fabrication. Resistor thicknesses <3 nm were excluded to avoid the unknown effects of hyper-thin W:VO_2_ films, with such properties not included within the scope of this work.

These designs were chosen to allow for the control of the overall material TCR response by varying the individual response of each component layer. As temperature increases from 0°C, the highest W-doped layer will transition into its metallic state first due to its lowest corresponding *T*_h_ value, decreasing the sample's overall resistivity. As the temperature increases further, subsequent layers will begin to transition in order based upon the W dopant percentage, decreasing the sample resistivity in a controlled manner. As such, the multi-layered stack should see a broadening of the TCR response compared to that of a single layered device.

Using a MOGA with NSGA-II technique (Figure S6), optimized designs were produced based upon two parameters, the stack's average TCR (*μ*) across the desired temperature window, and the associated standard deviation (*σ*) to quantify the flatness of the response. Figure S7 shows the resultant final Pareto frontier graphs of the multi-layered structured W:VO_2_ for both resistor geometries within the three selected temperature ranges. As the design structures are highly tailored to the chosen temperature window, achieving a particular design that performs well for all uses presents a challenge, and thus, the three windows were chosen to show the versatility of the model. Theoretically, for microbolometers, an ideal design needs the largest average TCR value as well as the smallest standard deviation, giving uniform high sensitivity. For multi-objective optimization, many results are available for selection, with the Pareto frontiers showing a strong inverse response. To assess the model’s effectiveness, the highest performing device, which we defined mathematically as: max (|μ|−σ) is presented together with the extremes of the Pareto front: the largest average TCR value and the smallest TCR standard deviation.

**Figure 6:**
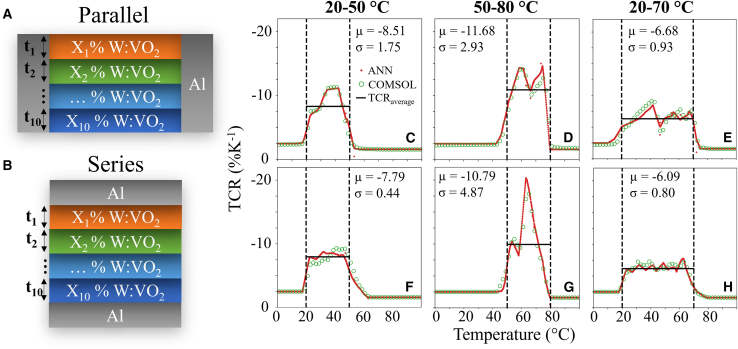
Simulated TCR response of multilayer W:VO_2_ The temperature dependent TCR response for the optimized designs found using the max (|μ|−σ) on the Pareto frontier. Schematic of (A) parallel and (B) series resistor geometries. Optimized TCR response for both resistor geometries: (C–E) parallel and (F–H) series. The results found using MOGA (red) are directly compared to simulations performed in COMSOL (green), with good agreement between the two techniques. The overlaid black dashed lines highlight the targeted operating temperature range for each design, with a solid horizontal line displaying the average TCR of each device to aid performance analysis.

The TCR performance of the optimized designs for both resistor geometries is presented in Figure 6. Calculated results are compared to responses simulated in COMSOL for the same stacks to ascertain the validity of the mathematical model. There is a good agreement between the two responses indicated by the closeness of the red and green circles (Figures 6C–6H). The slight discrepancy between models could be attributed to the difference between analytical calculation and finite element simulation. The MOGA model has successfully tailored the multi-layer W:VO_2_ stack designs to produce optimized responses based upon 2 objective parameters, with the average TCR overlaid on each plot in Figure 6. For the parallel resistor setup, the low temperature window design has an optimized average TCR of −8.51 (±1.75) %K^−1^. For commercial uses, a high TCR around room temperature is desired; as such, the low temperature window design can compete with the highest reported TCR values in literature.^35^^,^^68^

The optimized high and wide temperature range designs have associated average TCR values of −11.68 (±2.93) and −6.68 (±0.93) %K^−1^ respectively. These results show a performance that outperforms any reported wide-temperature VO_x_ based microbolometer in the literature, as shown in Table 1. The experimentally fabricated multilayer W:VO_2_ stack result is also presented in the table, similarly displaying a high TCR performance across an extended operating temperature range with an average heating TCR of −9.47 (±3.47) %K^−1^ between 30°C and 60°C. The simulated wide temperature range, high TCR design would allow for the extremely high accuracy detection of temperature changes seen in the absorbing material. These designs also consist of a simple planar stack, making them suitable for mass fabrication via an optimized ALD procedure, which offers nanometer precision and the ability to control W doping per layer by varying the cycle number of each material precursor. Similar results were seen in the series resistor geometries, generally with a slightly lower performance in both target parameters as shown in Figures 6F and 6H. Full design information for optimized stacks is presented in Tables S1 and S2 for resistors in parallel and series, respectively.

**Table 1:** Comparison of fabricated VO_x_ and WVO_2_ microbolometer designs deposited through various techniques, with estimated TCR and associated standard deviation across a sensor operation region as adjusted to be appropriate considering the resistivity response

Deposition method	Material	Design	Mean TCR (%/K)	Operational region (°C)	Film thickness (nm)	Reference
Pulsed laser deposition	VO_x_/WVO_x_	Multilayer W_x_V_1-x_O_2_	−10.5 (±0.2)	20 (22–42)	150	Émond et al.^37^
Magnetron sputtering	VO_x_	SiO_2_/SiN_4_/VO_x_	−2.8 (±0.4)	55 (25–80)	65	Dai et al.^41^
Magnetron sputtering	WVO_x_	Si/SiO_2_/WVO_x_	−1.8 (±0.6)	40 (30–70)	20	Zhang et al.^69^
RF magnetron sputtering	VO_2_(B)	5-stack Ti/MgF_2_ film/VO_2_	−1.6 (±0.3)	75 (25–100)	40	Lee et al.^70^
Reactive ion beam sputtering	VO_x_	SiO_2_/SiN_4_/VO_x_	−2.6 (±0.5)	55 (20–75)	70–150	Wang et al.^71^
Pulsed DC sputtering	α-VO_x_	Si_3_N_4_/VO_x_	−1.1 (±0.2)	75 (25–100)	60	Wang et al.^72^
Vapor transport	WVO_x_	Graded W-doped nanowires	−12.9 (±9.0)	37 (18–55)	–	Lee et al.^68^
Plasma enhanced ALD	VO_x_	Si_3_N_4_/Al:VO_x_/Al_2_O_3_	−2.8 (±0.5)	20 (30–50)	35	Kim et al.^73^
ALD	WVO_x_	Multilayer W:VO_2_ stack (experimental)	−9.5 (±3.5)	30 (30–60)	65	This work
ALD	WVO_x_	Multilayer W:VO_2_ stack (simulated)	−6.7 (±0.9)	50 (20–70)	440	This work

The wide temperature range multilayer W:VO_2_ response and the simulated optimized multilayer stack response results from these works are included for performance assessment.

Figure S8 displays the parallel resistor TCR performance of the selected designs from the Pareto Frontier of each temperature window. It is evident that for the points with the smallest variation, trivial solutions without TCR response can be generated. Similarly, the highest average TCR devices show poor optimization with a response essentially made from a single W:VO_2_ layer with a *T*_h_ occurring at the high end of the respective temperature window. The best performance was found when the average TCR and standard deviation were both considered. Similar trends were seen for the series resistor designs (Figure S9).

With these works, the optimized TCR response for any specified temperature window can be achieved using the simulated data and genetic algorithm techniques proposed, giving a prospective fast, low cost method for W:VO_2_ material development. Predictable control of the electric response of W:VO_2_ will provide an avenue for new technologies, from hyper-precise, super wide temperature thermal sensors, to memristor applications.^74^^,^^75^ Future works should focus on optimizing the IR radiation absorption of the active layer using more advanced designs not covered in the scope of this article, such as through the addition of a patterned gold black absorbing layer^76^ or depositing the stacks upon a Fabry-Perot quarter-wave cavity.^77^

### Conclusion

W:VO_2_ films of five different W dopant levels were successfully deposited via ALD and electrically characterized, giving a set of high-quality resistivity hysteresis measurements for each. These results were then parameterized using a model to produce a set of seven variables given by thirteen parameters to fully describe the temperature dependent change of resistivity in W:VO_2_ as a function of W at.%. The idea of using multiple discreet W:VO_2_ layers for a broadened phase transition response was investigated with the design limits made possible by ALD. As a proof of concept, two multilayer thin-films were fabricated consisting of discreet W:VO_2_ layers with individual doping percentages, with their material, electrical, and optical responses characterized. Successful W doping variation was evidenced using depth resolved XPS, with optimized annealed films displaying extended phase transition responses both electrically and optically, indicative of a multilayer response – experimental demonstration of a multilayer W:VO_2_ film deposited by ALD for a broadened phase transition. With an average heating TCR response of −9.5 (±3.5) %K^−1^ from 30°C to 60°C, such materials show great promise as high sensitivity detecting layer.

To push the limits of such a multilayer W:VO_2_ design, a state-of-the-art MOGA with NSGA-II technique was implemented using the newly modeled W:VO_2_ resistivity responses to produce optimized designs for three temperature windows based upon two parameters: the average TCR and associated standard deviation. Resulting Pareto frontiers were analyzed giving a wide range of optimized designs with max (|μ|−σ) as excellent compromise. Planar W:VO_2_ multilayer stacks were simulated with an average TCR value of −6.7 (±0.9) %K^−1^ over a wide temperature range of 20°C–70°C, −11.7 (±2.9) %K^−1^ across a high temperature window from 50°C to 80°C, and −8.5 (±1.8) %K^−1^ from 20°C to 50°C. Such material performances allow extended operating regions, which is attractive for high sensitivity microbolometers, including both amplitude modulated, and frequency modulated designs.

### Limitations of the study

It is worth noting that the simulated W-doped VO_2_ electrical responses are based upon our experimental ALD results; thus, W-doped VO_2_ produced via other deposition methods may have different responses. We expect our model to keep its validity, though with different parameters. A deeper understanding of the mechanisms behind the characterized electrical responses would require additional work. Finally, this study comparison focuses on TCR material performance; a follow up investigation would be required to assess the performance of a real uncooled microbolometer device using WVO_2_ multilayers.

## Resource availability

### Lead contact

Additional inquiries and requests for resources and information should be directed to and will be fulfilled by the lead contact, C.H. (Kees) de Groot (chdg@soton.ac.uk).

### Materials availability

This study did not generate new unique reagents.

### Data and code availability

• Date reported in this article is available from https://doi.org/10.5258/SOTON/D2577.
• The original code is available from https://doi.org/10.5258/SOTON/D2577.
• Any additional information required to reanalyze the data reported in this article is available from the lead contact upon request.

## Acknowledgments

This work was supported by the Research Council U.K. (10.13039/501100000690RCUK) under grant EP/VO62689/1. This project has also received funding from the European Union’s 10.13039/501100007601Horizon 2020 research and innovation program under Grant Agreement No. 821932 “SMART-FLEX.”

## Author contributions

Conceptualisation, C.W., C.H.D.G., O.L.M., and K.S.; methodology, C.W., K.S., and Y.Z.; formal analysis, C.W., K.S., and Y.Z.; investigation, C.W., K.S., B.D., and Y.Z.; writing – original draft, C.W., writing – review and editing, C.W. C.H.D.G., and K.S.; funding acquisition, C.H.D.G. and O.L.M.; supervision, C.H.D.G., K.S., O.L.M., and R.H.

## Declaration of interests

The authors declare no conflict of interest.

## STAR★Methods

### Key resources table

**Table:** 

REAGENT or RESOURCE	SOURCE	IDENTIFIER
**Chemicals, peptides, and recombinant proteins**		

Tetrakis(ethylmethylamino)-vanadium(IV) 98%	STREM	CAS: 791114-66-4
Tungsten hexacarbonyl (W(CO)_6_) 99%	STREM	CAS: 14040-11-0

**Software and algorithms**		

COMSOL Multiphysics 6.0	Comsol, Inc.	www.comsol.com
Avantage V6	ThermoFischer	www.thermofisher.com
MOGA code	University of Southampton	https://doi.org/10.5258/SOTON/D2577

### Method details

#### Device fabrication

Tetrakis(ethylmethylamino)-vanadium(IV) (TEMAV) 98% and Tungsten hexacarbonyl (W(CO)_6_) 99% were used as the Vanadium and Tungsten precursors in the ALD system, respectively. Both precursors were oxidised using deionised water. Films were deposited on Si wafers coated with 100 nm Al and 1100 nm SiO_2_ at 200°C in a Savannah S200 ALD system. For W dopant levels, V/W cycle ratios were used of 0, 0.2, 0.6, 0.8 and 1.2 with V cycle number set to 880 for 40 nm thick films.^48^ To crystallise the deposited films into VO_2_ (M1), a post deposition anneal was performed in an Oxford instruments Agile NanoFab CVD system. Optimised anneal conditions were 400°C for 2 hours with oxygen partial pressure of 133 Pa. The multilayer W:VO_2_ thin-film was deposited on a Si wafer coated with the same underlying structure as the single layer films (80 nm Al/1100 nm SiO_2_). To produce discreet W:VO_2_ layers with individual doping concentrations, multiple super cycles were combined with varying V/W ratios. The W at.% was increased from the bottom upwards. A full anneal study was conducted on cleaved chips to produce the best electrical performance.

#### Device characterization

##### X-ray photoelectron spectroscopy (XPS)

Surface XPS measurements were performed on as-deposited and annealed multilayer W:VO_2_ sample using a Thermo Scientific Theta Probe X-ray photoelectron spectroscopy system in ultrahigh vacuum conditions (base pressure P ≈ 1.0 × 10^−7^ Pa). An electron flood gun was used during data collection to reduce surface charging effects. The XPS data was analysed using o Avantage software. Depth resolved XPS measurements were conducted on the as-deposited multilayer W:VO_2_ samples using the same conditions as the surface measurements to ensure accurate comparison of the results. A 3 keV Ar^+^ ion etch was used to sputter material between measurements, giving a depth resolved XPS profile. The ion beam was incident on the material surface at an angle ≈75°, producing an etch crater with ≈2 mm diameter, much larger than the spot size of the x-ray gun to minimise signal from the etched holes wall. 20 etch levels measurement with an Ar^+^ etch time of 15 seconds between them were performed on each sample. Full etch rate analysis and further details of the fitting procedure for both surface and depth resolved measurements can be found in the supplemental information.

##### Van der Pauw measurements

A Nanometrics H 5550 was used to collect temperature dependent v/d Pauw measurements, with sample temperature controlled by liquid nitrogen and a heater. Spring-loaded metallic contacts provided high quality ohmic contact between the samples and measurement tool. Resistance values were taken 1 minute after reaching the target temperature, with the films measured under 1.33 × 10^-2^ Pa vacuum to prevent condensation on the sample surface during cooling. Temperature sweeps were performed by cool down and subsequent heat up.

##### Raman spectroscopy

Room temperature Raman spectroscopy measurements were performed on both as-deposited and post annealed samples using a Renishaw inVia laser Raman spectrometer with a 532 nm laser. Laser power was kept below 0.1 mW at the sample surface to avoid film heating during measurements, with all samples measured under atmospheric conditions. All samples were cooled to 3°C before any measurements to ensure they were in their low-temperature monoclinic phase. It is also worth noting that Raman spectroscopy is a surface due to blue light penetration depth of around 30 nm in VO_2_ in its insulating phase,^78^ which is our samples not showing the strong SiO_2_ substrate peak around 520cm^-1^.^66^

##### Fourier transform infrared spectroscopy (FTIR)

The infrared (IR) reflectance and transmission were measured in the range 2.5 – 20 μm using a Thermo-Nicolet Nexus 670 with a continuum microscope and 15x optical objective with a numerical aperture of 0.58. Liquid nitrogen cooled MCT detector with a 100x100 μm spot size was used, along with a far IR light source and KBr beam splitter. The spectrometer was left to cool for 2 hours to reach a stable temperature, which is maintained for around 5 hours until the liquid nitrogen evaporates. An aluminium mirror in air was used as a highly reflective reference for all reflection measurements.

##### Computational methods

A multi-objective genetic algorithm (MOGA) with NSGA-II (Non-dominated Sorting Genetic Algorithms) technique is used to find the optimised microbolometer designs within a Python environment. The optimisation of TCR is comprised of two variables, the average TCR and its standard deviation. Full details can be found in the supplemental information. *COMSOL* was used to verify the electrical response of various planar VO_2_/W:VO_2_ stacks found using the NSGA-II technique from 0-100 °C. The models consist of a 10-layer design for resistors in parallel and series, with each layer having an individual W at.%. Two aluminium electrodes are attached to the sides of the VO_2_ layers, with a constant 5V applied between them to measure the change of resistance across the stack, with heating applied uniformly across all W:VO_2_ layers. Layer thicknesses were constrained to be between 3 – 100 nm each, such that the stacks remain physical and could be fabricated via ALD, while not including unknown hyper-thin film effects.

### Quantification and statistical analysis

There are no quantification or statistical analyses to include in this study.
