# Supplementary material for: Multilayer W-doped vanadium dioxide thermal sensors with extended operation region
Source: iScience. 2025 Apr 24;28(6):112528. doi: 10.1016/j.isci.2025.112528 (PMC12225922; doi:10.1016/j.isci.2025.112528)
Supplement: Document S1. Figures S1–S9 and Tables S1 and S2 [file mmc1.pdf]

iScience, Volume *28*

Supplemental information

Multilayer W-doped vanadium dioxide thermal sensors with extended operation region

Callum Wheeler, Yuxiao Zhu, Kai Sun, Bohao Ding, Ruomeng Huang, Otto L. Muskens, and C.H. (Kees) de Groot

A. X-Ray Diffraction

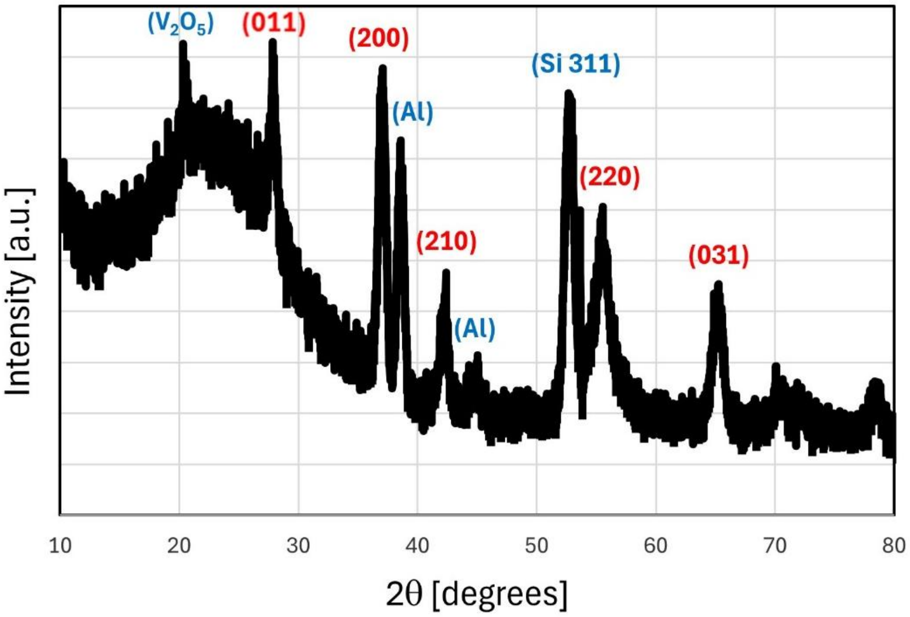

Figure S 1: Grazing incidence X-Ray Diffraction of undoped VO_2 film. X-ray diffraction data were taken under grazing incidence on a Rigaku Smartlab thin film diffractometer. The resulting data for a undoped VO_2 film after annealing. All major peaks are indexed by the monoclinic (low temperature) VO_2 phase (JCPDS No. 43-1051). A small peak related to V_2O_5 is visible at low angle. Other peaks are related to the Si substrate, where the grazing angle reflect on the shoulder of the 311 surface of the Si single crystal, and the Al layer which is buried in the stack for optical radiative cooling measurements.

B. Electrical characterisation

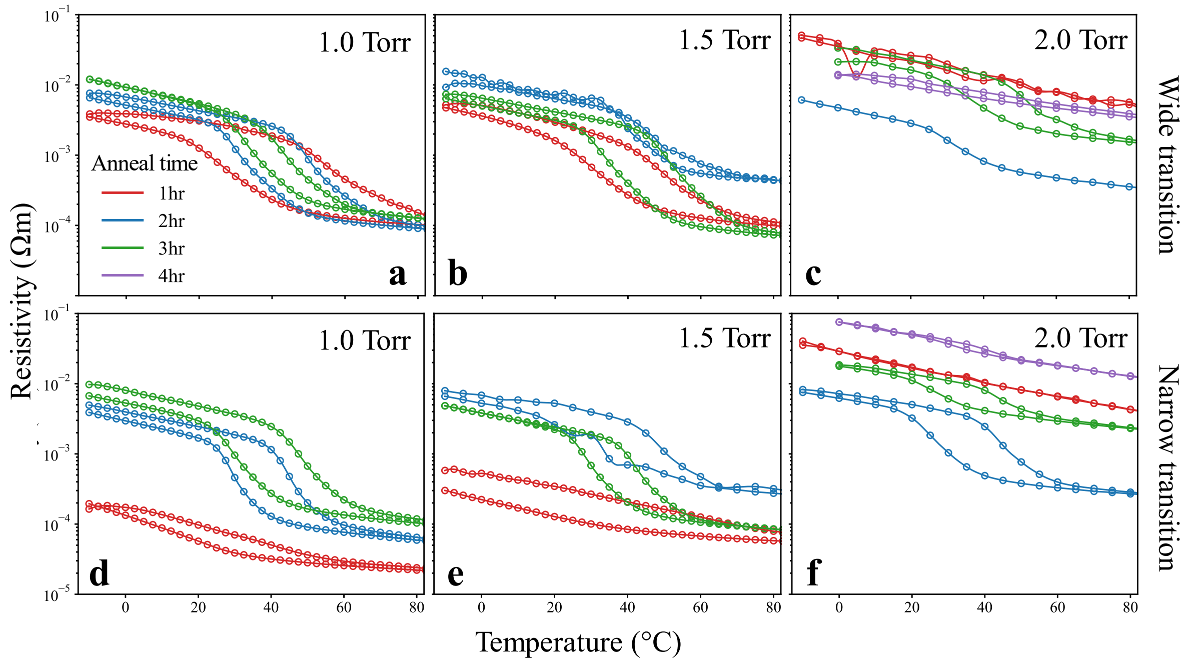

Figure S 2: The temperature-dependent resistivity response of all annealed multilayer W:VO_2 samples. The (a-c) wide band and (d-f) narrow band sample responses are presented as a function of anneal pressure and anneal time for heating and cooling runs.

C. **Optical characterisation**

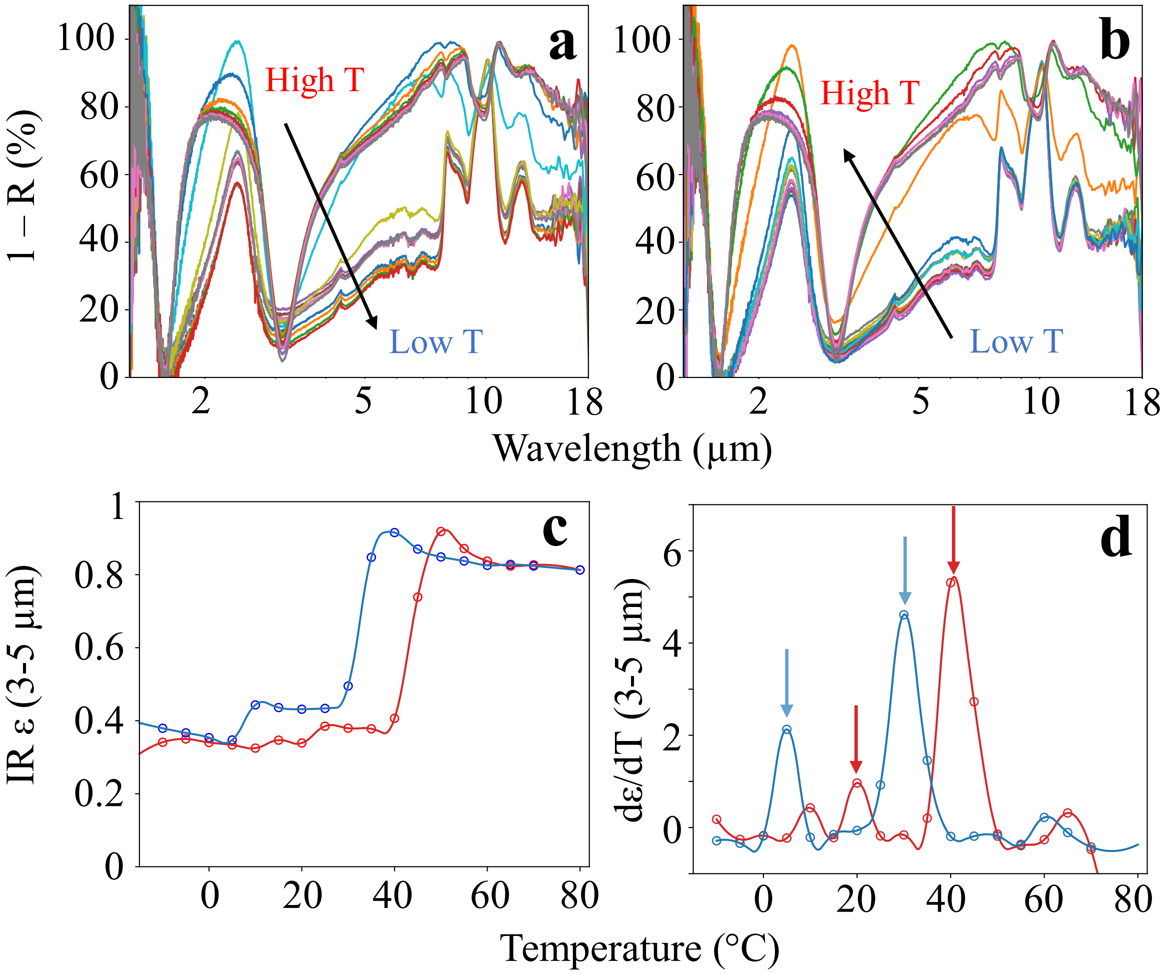

Figure S 3: The temperature dependent optical response of the optimised multilayer device. The raw reflection spectra measured at discreet temperatures from -10 to 80 °C for (a) cooling and (b) heating runs. Due to the nature of the meta-reflector stack using interference to enhance absorption, there is an oscillatory pattern in the 1-R spectra, with peaks seen at ≈1.1 and 2.3 μm corresponding with the thickness of the dielectric spacer. (c) The integrated blackbody weighted emissivity response of the multilayer device for heating and cooling runs. (d) The derivative of the emissivity vs sample temperature with two peaks highlighted, corresponding to individual W:VO_2 layers transitioning at different temperatures.

D. Extraction of model resistivity response of W:VO_2

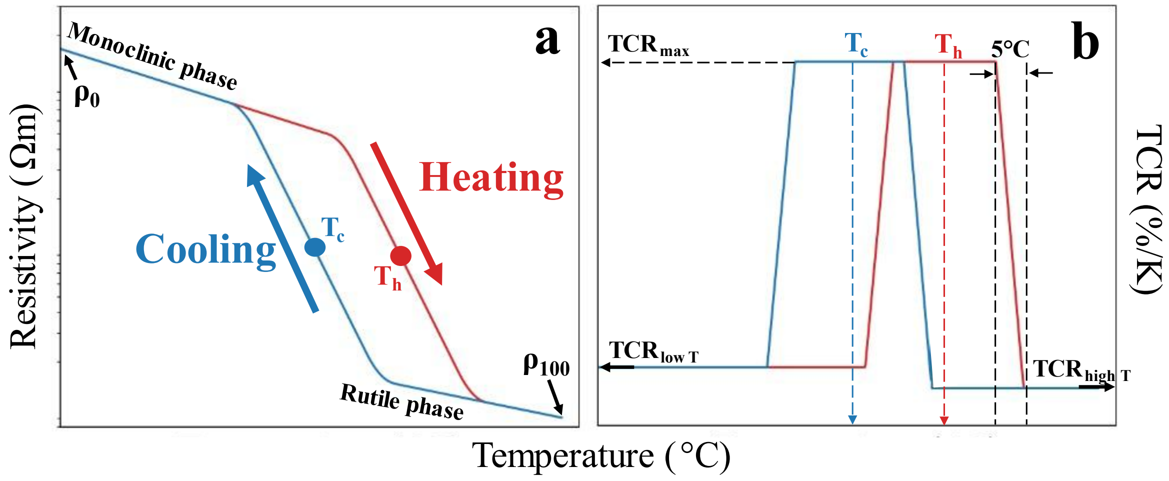

Figure S 4: The proposed resistivity fitting model for W:VO_2 thin films, including both the (a) resistivity and (b) TCR response. The main parameters used to quantify this response are also labelled including: ρ_0, ρ_{100}, T_c, T_h, $TCR_{low\ T}$, $TCR_{high\ T}$, and TCR_{max}.

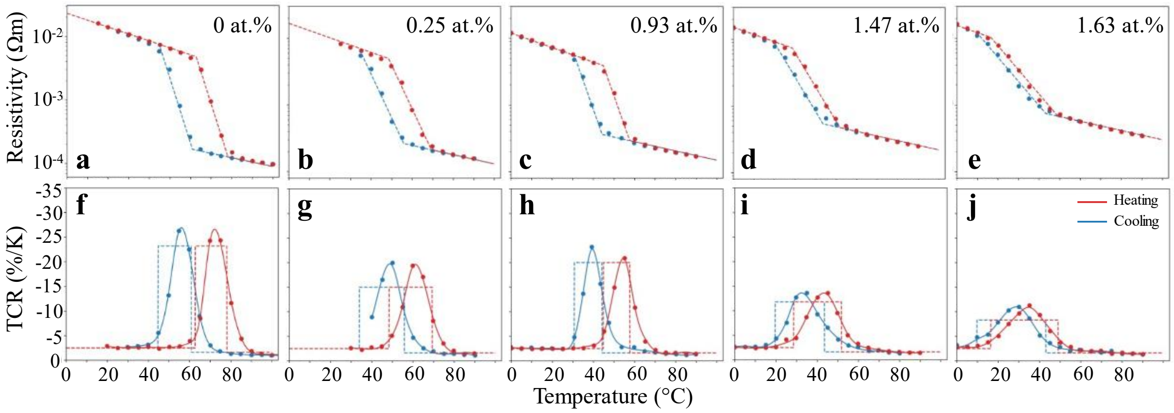

Figure S 5: Parameter extraction from W:VO_2 thin film electrical performance. (a-e) Optimised resistivity fits using the new proposed model on experimental data to extract the parameters given in Table 1. (f-j) The associated TCR response for each at.% is shown directly below the resistivity response, with dashed lines being the predicted response fit, and solid line a spline fit to experimental data (dots) for heating and cooling runs.

E. MOGA method

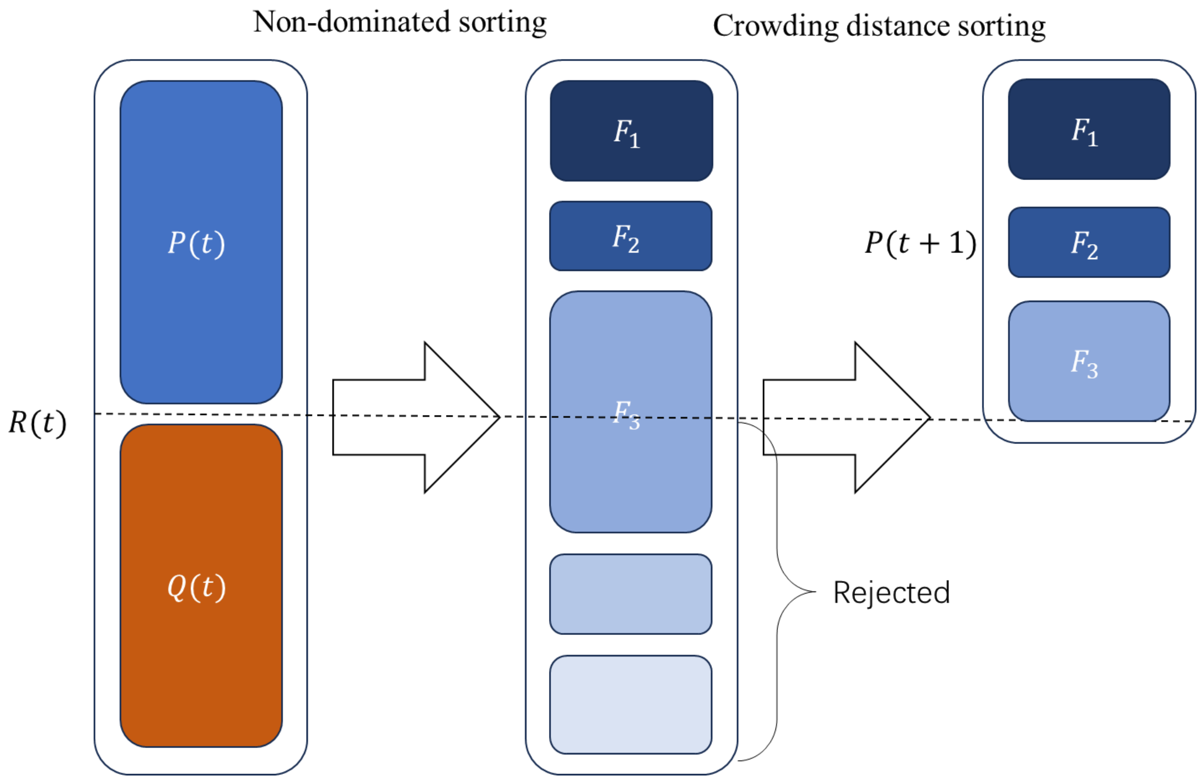

Figure S 6: Flow chart of multi-objective genetic algorithm used in the present study, where P(t) is the parent population with size N, Q(t) is the new offspring population, and R(t) is the child population resulting from P(t) and Q(t). A fast non-dominated sorting algorithm creates non-dominated fronts F_1, F_2,… F_k in $R(t)$. The crowding distance of the solutions in identified fronts is calculated. Sequentially F_1, F_2,… F_k are placed into $P(t+1)$, until the size of $P(t+1)$ exceeds the population size limit N when a certain layer F_j is reached. $P(t+1)$ continues to be filled according to the order of decreasing crowding distance of individuals in F_j until the population size reaches N.

F. Pareto frontier

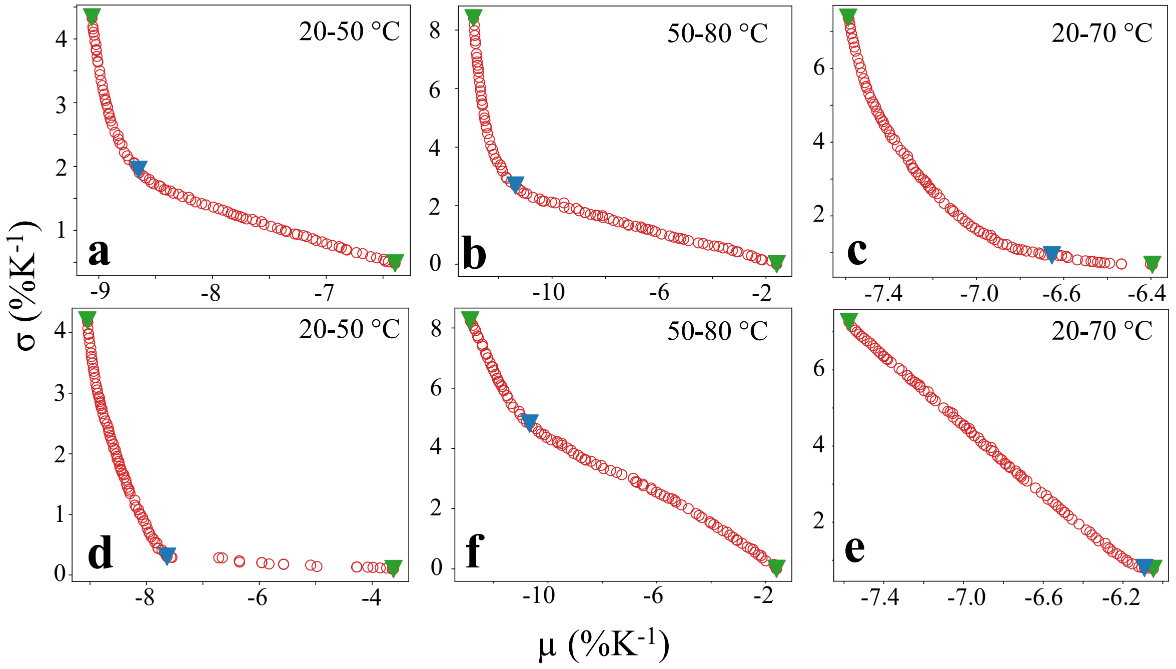

Figure S 7: The Pareto frontier plots produced using the MOGA with NSGA-II technique in a Python environment. Results for three targeted operating temperature windows are presented for both simulated resistor geometries – (a-c) parallel and (d-f) series. 100 optimised designs for each were produced using the MOGA with NSGA-II technique based upon two parameters – the stacks average TCR (μ) across the desired temperature window, and the associated standard deviation (σ) to quantify the flatness of the response. The green triangles represent the extreme designs with max average TCR or lowest standard deviation. The blue triangles represent max (|μ|-σ) and are considered the best compromise.

G. Temperature dependent TCR response of optimised designs

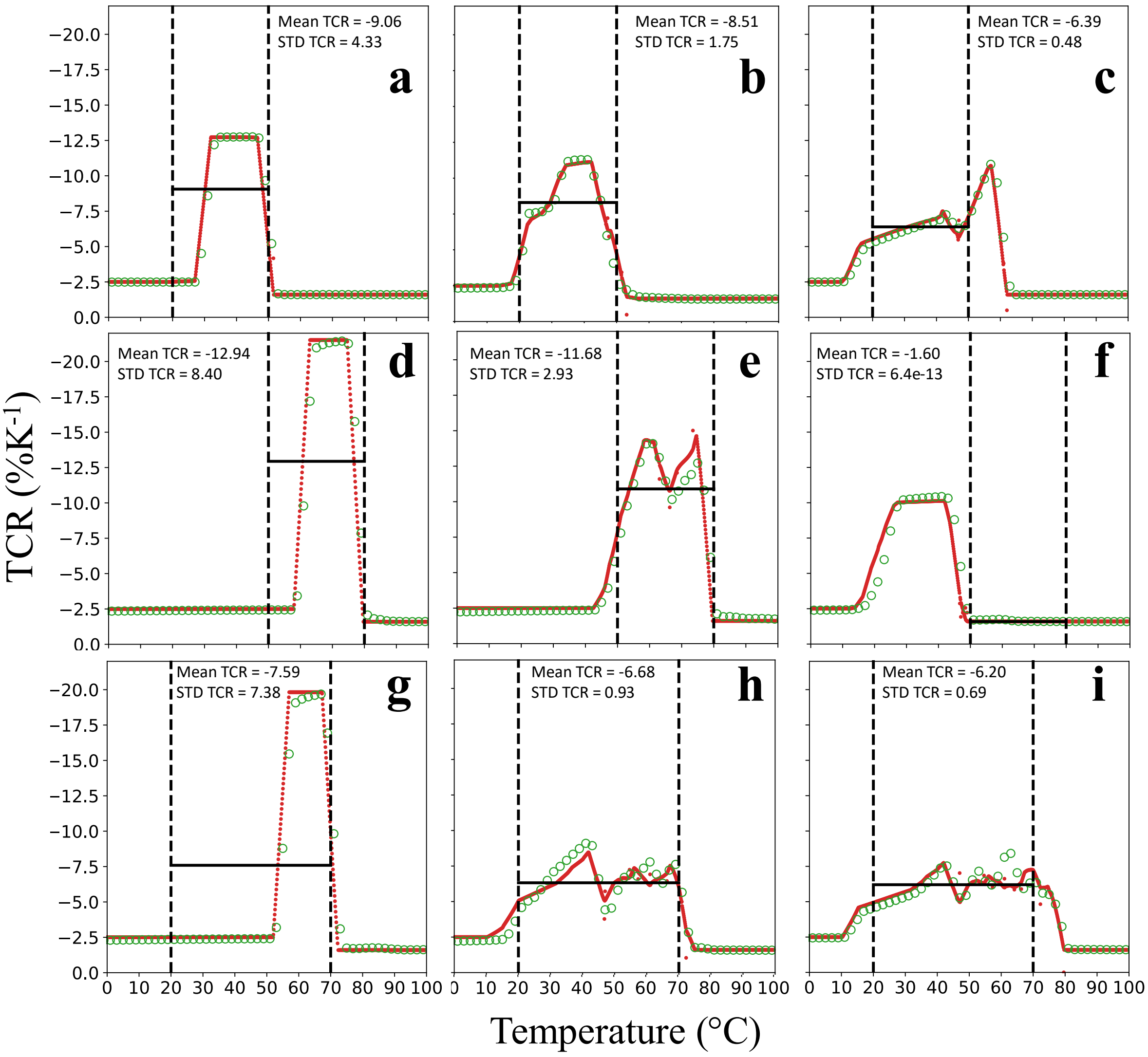

Figure S 8: Resistors in parallel optimised designs chosen from the Pareto frontier for three respective temperature windows: 20 – 50 (low), 50 – 80 (high), and 20 – 70 (wide) °C, with this information on each plot as a black dashed line. (a,d,g) The highest TCR designs, (b, e, h) the max(abs(μ)-σ) optimised designs, and (c, f, i) the lowest standard deviation designs. A direct comparison between the python results (red) and COMSOL (green) simulations for the same designs are overlaid on each plot, with the average TCR shown as a straight black line.

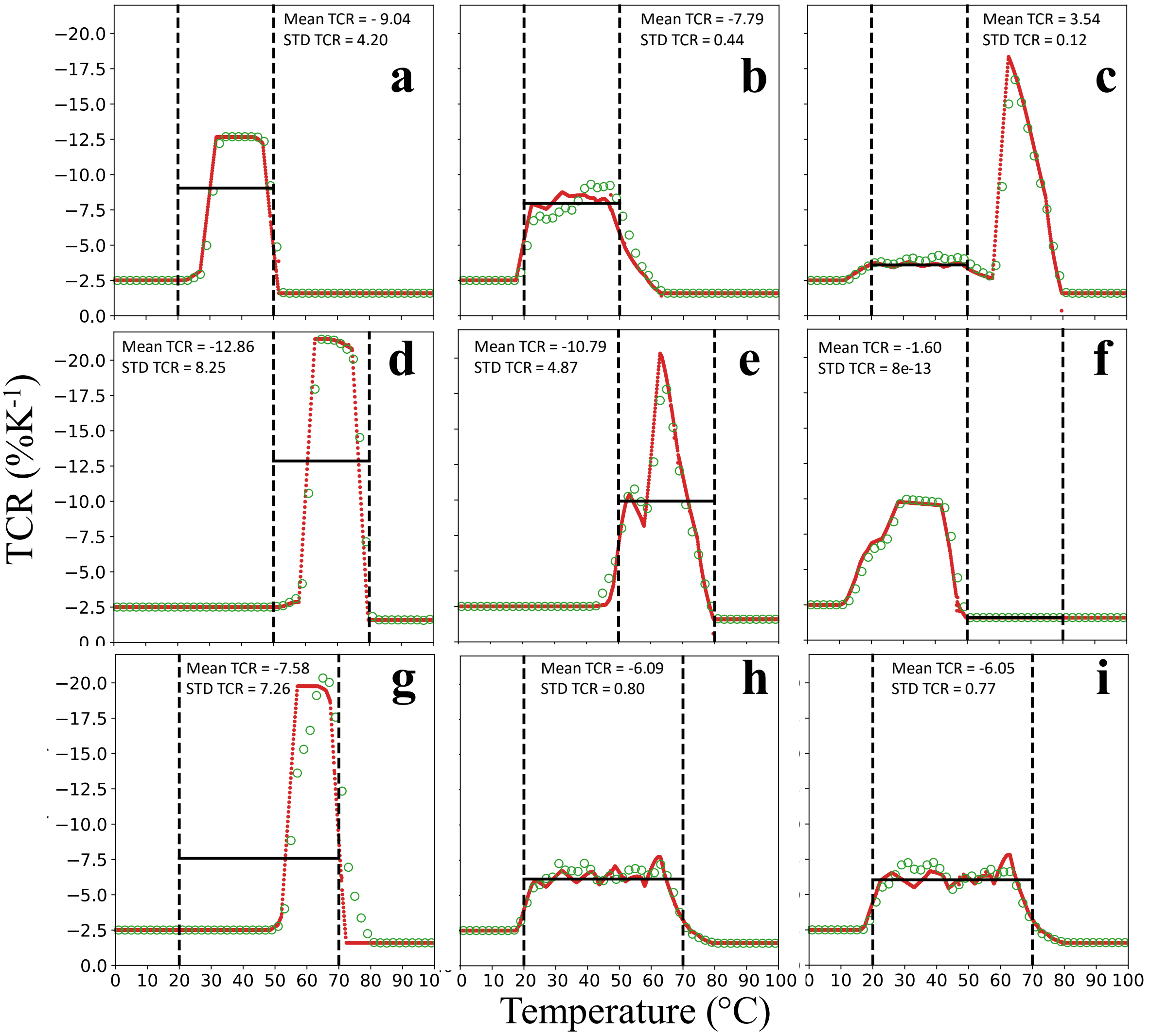

Figure S 9: Resistors in series optimised designs chosen from the Pareto frontier for three respective temperature windows: 20 – 50 (low), 50 – 80 (high), and 20 – 70 (wide) °C, with this information on each plot as a black dashed line. (a, d, g) The highest TCR designs, (b, e, h) the max(abs(μ)-σ) optimised designs, and (c, f, i) the lowest standard deviation designs. A direct comparison between the python results (red) and COMSOL (green) simulations for the same designs are overlaid on each plot, with the average TCR shown as a straight black line.

Layer	a		b		c		d		e		f		g		h		i	
	W	T	W	T	W	T	W	T	W	T	W	T	W	T	W	T	W	T
	at.%	nm	at.%	nm	at.%	nm	at.%	nm	at.%	nm	at.%	nm	at.%	nm	at.%	nm	at.%	nm
1	0.25	67	1.75	47	0.75	47	1.3	21	1.2	23	1.95	14	0	24	0	21	1.75	23
2	0.25	62	0.3	39	0.5	30	1.65	3	1.65	5	1.9	9	0	96	0	88	1.85	79
3	0.25	33	0.9	78	0.95	19	1.3	96	1.65	29	1.95	89	0.45	3	0.4	13	1.55	10
4	0.25	68	0.6	72	0.6	16	1.3	3	1.2	34	0.8	3	0	87	0	92	1.65	27
5	0.25	86	1.1	10	2	93	1.3	83	1.2	41	0.75	41	0.75	3	0.65	40	0.65	3
6	0.25	53	0.25	39	0.25	46	1.3	45	1.7	25	2	46	0	100	0	19	1.75	95
7	0.25	5	1.8	100	1.1	27	1.3	3	1.85	9	0.7	84	0.7	3	0.5	53	1.9	14
8	0.25	92	0.2	34	0.15	39	1.3	73	1.7	93	0.7	72	0	54	0	35	1.55	97
9	0.25	3	0	3	0	82	1.3	3	1.0	4	0.7	98	0	73	0.2	42	2	3
10	0.25	34	0.25	46	2	41	1.3	72	1.3	32	2	68	0	96	0	55	1.5	55

Table S1: Full design information for optimised resistors in parallel MOGA stacks presented in **Figure S 8**. W is layer tungsten doping atomic %, and T is individual layer thickness.

Layer	a		b		c		d		e		f		g		h		i	
	W	T	W	T	W	T	W	T	W	T	W	T	W	T	W	T	W	T
	at.%	nm	at.%	nm	at.%	nm	at.%	nm	at.%	nm	at.%	nm	at.%	nm	at.%	nm	at.%	nm
1	0.3	27	1.65	76	1.05	43	1.3	12	0.9	9	0	94	0.2	10	0.4	5	1.45	63
2	0.25	70	0.6	57	0.6	50	1.5	14	1.6	19	1.95	100	0.4	3	0.45	65	1.8	34
3	0.3	43	1.7	75	1.7	73	1.5	3	1.7	35	0	76	0	16	0	58	2	42
4	0.25	85	1.65	72	1.75	79	1.3	3	1.75	9	1.75	76	0	86	0.5	34	1.9	57
5	0.25	90	0.25	22	0.25	22	1.3	88	1.75	91	0.65	35	0.25	7	0.25	11	1.95	44
6	0.25	80	0.45	12	0.4	13	1.35	6	1.45	10	0.9	12	0	33	0.4	97	1.8	42
7	0.25	39	1.7	45	1.7	75	1.3	49	1.2	11	1.3	46	0	16	0	3	1.95	35
8	0.25	91	1.3	74	1.55	74	1.3	100	0.65	31	0	88	0	58	0	3	1.45	9
9	0.4	3	0	23	0	22	1.45	7	1.8	60	0.6	13	0	68	0.65	3	1.85	15
10	0.4	26	0.9	39	0.9	15	1.45	3	1.75	31	0.95	15	0	99	0	20	1.65	33

Table S2: Full design information for optimised resistors in series MOGA stacks presented in **Figure S 9**. W is layer tungsten doping atomic %, and T is individual layer thickness.
